# Development of a small animal model for deer tick virus pathogenesis mimicking human clinical outcome

**DOI:** 10.1371/journal.pntd.0008359

**Published:** 2020-06-15

**Authors:** Meghan E. Hermance, Charles E. Hart, Allen T. Esterly, Erin S. Reynolds, Jahnavi R. Bhaskar, Saravanan Thangamani

**Affiliations:** 1 SUNY Center for Environmental Health and Medicine, SUNY Upstate Medical University, Syracuse, New York, United States of America; 2 Institute for Global Health and Translational Sciences, SUNY Upstate Medical University, Syracuse, New York, United States of America; 3 Department of Microbiology and Immunology, SUNY Upstate Medical University, Syracuse, New York, United States of America; University of Florida, UNITED STATES

## Abstract

Powassan virus (POWV) is a tick-borne flavivirus that encompasses two genetic lineages, POWV (Lineage I) and deer tick virus (DTV, Lineage II). In recent years, the incidence of reported POWV disease cases has increased, coupled with an expanded geographic range of the DTV tick vector, *Ixodes scapularis*. POWV and DTV are serologically indistinguishable, and it is not known whether clinical manifestations, pathology, or disease outcome differ between the two viruses. Six-week-old male and female BALB/c mice were footpad-inoculated with DTV doses ranging from 10^1^ to 10^5^ FFU. Dose-independent mortality, morbidity, and organ viral loads were observed for mice inoculated with sequentially increasing doses of DTV. By study completion, all surviving mice had cleared their viremias but detectable levels of negative-sense DTV RNA were present in the brain, indicating viral persistence of infectious DTV in the central nervous system. For mice that succumbed to disease, neuropathology revealed meningoencephalitis characterized by microscopic lesions with widespread distribution of viral RNA in the brain. These findings, coupled with the rapid onset of neurological signs of disease and high viral titers in nervous tissue, highlight the neurotropism of DTV in this mouse model. Additionally, disease outcome for DTV-infected mice was not affected by sex, as males and females were equally susceptible to disease. This is the first study to comprehensively characterize the clinical disease outcome in a small animal model across a spectrum of POWV/DTV infection doses. Here, we developed a small animal model for DTV pathogenesis that mimics the manifestations of POWV disease in humans. Since it is currently not known whether DTV and POWV differ in their capacity to cause human disease, the animal model detailed in our study could be utilized in future comparative pathogenesis studies, or as a platform for testing the efficacy of vaccines, and anti-virals.

## Author summary

Powassan virus (POWV) is a tick-borne flavivirus that can result in a fatal encephalitic disease in humans. Although POWV is closely related to deer tick virus (DTV), each virus is maintained in nature by unique transmission cycles. Because POWV and DTV are serologically indistinguishable and must be differentiated by genetic sequence analysis, an unknown portion of previously reported POWV cases may have actually been DTV cases. Overall, the scientific literature is sparse when it comes to describing confirmed DTV cases or the neurovirulence and clinical outcome for DTV-infected mice. Our study is the first to comprehensively characterize the survival rates, timeline of clinical disease, and tissue distribution of virus for mice infected with a range of DTV doses. Signs of disease in the DTV-infected mice mimicked the course of human clinical disease where neurological manifestations were preceded by a generalized febrile illness. Sequentially increasing doses of DTV did not elicit increased mortality rates or organ viral loads, which suggests that in this mouse model, virus infection and replication in tissues is independent of the infection dose. Our findings support the use of this mouse model in future DTV pathogenesis studies.

## Introduction

Compared to other blood-feeding arthropods, ticks transmit the largest variety of human and domestic animal pathogens, including bacteria, protozoa, and viruses [1]. Tick-borne diseases accounted for >75% of human vector-borne disease cases reported to the United States National Notifiable Diseases Surveillance System between 2004 to 2016 [2]. In recent decades, several tick-borne viruses have emerged as human and veterinary health concerns across the globe, including Tick-borne encephalitis virus (TBEV), Crimean Congo hemorrhagic fever virus, Alkhumra hemorrhagic fever virus, Kyasanur Forest virus, African swine fever virus, and Severe Fever with Thrombocytopenia virus, as well as several that are specific to North America, including Powassan virus (POWV), Heartland virus, and Bourbon virus.

POWV is the only autochthonous tick-borne flavivirus in North America, and increasing numbers of human POWV cases have been detected over the past 20 years [3–5]. POWV was first discovered in 1958 when a five-year-old boy succumbed to a severe encephalitic disease in the town of Powassan, Ontario [6]. Human cases of POWV have since been reported in the northeastern and upper midwestern United States and in eastern Canada. In humans, POWV infections can be asymptomatic or can result in a severe neuroinvasive disease that clinically presents as encephalitis, meningoencephalitis, or aseptic meningitis. The case fatality rate is approximately 10%, and long-term neurological sequelae may arise in 50% of POWV survivors [4]. Neuroinvasive POWV became a nationally notifiable disease condition in 2001 in the United States. From 1958 through 1998, only 27 human cases of POWV were reported between the United States and Canada combined [3]; however, in the United States alone, 188 cases of POWV disease have been reported from 2004 to 2019 [2, 7]. The increase in the number of reported human POWV disease cases over the past fifteen years could be attributed to increased arthropod-borne virus surveillance following the 1999 introduction of West Nile virus into North America, increased awareness of POWV disease among clinicians and diagnosticians, changes in tick vector species and/or vertebrate host ecology, or a combination of these factors [4, 8].

Two genetic lineages exist for POWV: Lineage I includes the prototypic POWV LB strain isolated from the index case [6], while Lineage II consists of deer tick virus (DTV), which was first isolated from *Ixodes scapularis* ticks [9]. Although these two lineages of POWV share approximately 84% nucleotide sequence identity and 94% amino acid identity, they are serologically indistinguishable and constitute two genotypes of the same virus [4, 10–11]. Phylogenetics indicate that natural selection-driven divergence of the two lineages is likely attributed to the close association of each lineage with specific tick vector species in nature [12]. In North America, there appears to be three main enzootic maintenance cycles of POWV. POWV is transmitted between *Ixodes cookei* ticks and groundhogs / mustelids as well as between *Ixodes marxi* and squirrels, while DTV is maintained between *Ixodes scapularis* and white-footed mice [9, 13–14]. *I*. *cookei* and *I*. *scapularis* exhibit different host-seeking behaviors. Unlike *I*. *cookei*, *I*. *scapularis* are not as host-specific, are non-nidicolous, and are more likely to aggressively parasitize a variety of hosts, including white-footed mice, deer, and occasionally humans [3–4].

The development of an appropriate animal model of arthropod-borne viral disease in humans is imperative for the evaluation of therapeutics and vaccine candidates. Laboratory strains of mice have been extensively used for modelling flavivirus encephalitic disease because they recapitulate many of the neurological disease syndromes observed in humans. Immunocompetent strains of mice, including BALB/c and C57BL/6, develop a generalized febrile illness and neurological disease when peripherally dosed with POWV [15–17]. In 3 to 6-week-old BALB/c mice infected with POWV (LB strain) via footpad or intraperitoneal inoculation, the first clinical signs of febrile illness, including ruffled fur, hunched posture, weight loss, and lethargy, are typically observed between 5 to 6 days post-infection (dpi) [15–16, 18]. Following the initial febrile illness, juvenile BALB/c mice can rapidly progress to neurological disease, including paresis, ataxia, hind limb paralysis, or flaccid paralysis, with up to 100% mortality [15–16, 18]. In juvenile to young-adult C57BL/6 mice infected with POWV (LB strain) via footpad or intraperitoneal inoculation, similar signs of disease are observed, beginning as early as 5–6 dpi, with mortality rates ranging from 60–100% [17–19]. It has been suggested that the differences in POWV mortality rates observed between studies that utilized the same strain of mice could be attributed to routes of inoculation, the source of mice, or genetic differences in the virus stock itself [18].

Compared to POWV, few publications detail neurovirulence and clinical outcome in mice infected with DTV. DTV was initially suggested to be less virulent than other members of the tick-borne encephalitis virus complex [9]; however, when 5 to 6-week-old female NIH Swiss mice were intraperitoneally inoculated with DTV (IPS-001 strain) or POWV (LB strain), the LD_50_’s were comparable (10^2.7^ PFU and 10^2.3^ PFU, respectively), with the onset of disease only slightly delayed in DTV-infected mice [10]. When DTV (Spooner strain)-infected *I*. *scapularis* nymphs were fed on 6-week-old BALB/c mice for at least 30 minutes, 100% of mice became infected, and clinical signs of disease, ranging from ruffled fur and weight loss to weakness, ataxia, and hind limb paralysis, were observed between 5–11 days post-tick attachment [20]. In a recent study, when 7 and 14-week-old C57BL/6 mice were footpad inoculated with 10^2^ focus-forming units (FFU) of DTV- Spooner, mortality rates were ~ 60% [19].

No study has comprehensively characterized the mortality, morbidity, and neuropathology as related to a range of POWV or DTV infection doses. In the present study, we sought to develop a small animal model of DTV pathogenesis that mimics the human clinical outcome of disease. We investigated the morbidity, mortality, viral loads, and neuropathology associated with five infection doses of DTV (ranging from 10^1^–10^5^ FFU) and whether sex affects disease outcome in BALB/c mice.

## Materials and methods

### Ethics statement

All experiments involving mice were conducted in animal biosafety level 3 (ABSL-3) facilities in strict accordance with an animal use protocol approved by the University of Texas Medical Branch (UTMB) Institutional Animal Care and Use Committee (IACUC: # 0907054).

### Cells and viruses

African green monkey kidney (VeroE6) cells were purchased from the American Type Culture Collection (ATCC) and maintained in culture with Modified Eagle’s Medium (MEM) supplemented with 10% fetal bovine serum (FBS), 1% non-essential amino acids, and a 1% antibiotic mixture of penicillin/streptomycin incubated at 37°C with 5% CO_2_. The World Reference Center for Emerging Viruses and Arboviruses at UTMB provided stock of the Spooner strain of DTV, which had previously been passaged 1 time in suckling mice brains. The stock was then passaged 6 times on VeroE6 cells. Stock virus titers were determined by focus-forming immunoassay as described previously [21]. Next generation sequencing demonstrated that the consensus nucleotide sequence of the DTV genome was 99.84% identical the DTV Spooner strain. 17 nucleotide sequence differences were observed between our DTV Spooner strain stock and that of the DTV Spooner, Wisconsin isolate (GenBank: HM440560.1), which resulted in the following 3 amino acid sequence differences in: M (N213K), NS4B (S2368T), and NS5 (K2903R).

### Animals and inoculations

Five-week-old female and male BALB/cj mice were obtained from The Jackson Laboratory (Bar Harbor, ME). Mice were acclimated to the local environment before initiation of experiments, at which point the mice were six weeks of age. Mice were housed in groups of 4 by gender in individually ventilated cage systems with controlled humidity, temperature, and light (12:12 light-dark cycle). Food and water were available *ad libitum*.

Footpad inoculations were performed under isoflurane anesthesia with DTV doses ranging from 10^1^ focus-forming units (FFU) to 10^5^ FFU in 30 μL of serum-free MEM media. A sterile 29-gauge 0.5-inch needle was used for each inoculation. Mock-infected control mice were footpad inoculated with equivalent amounts of serum-free MEM media. Daily body weights and clinical observations were documented for each mouse. Retro-orbital bleeds were performed under isoflurane anesthesia on alternating days through 6 dpi (males were bled at 0, 2, 4, and 6 dpi; females were bled at 1, 3, and 5 dpi). Blood was harvested via terminal cardiac puncture during necropsies. A clinical scoring sheet (S1 Table) was used to assess appearance (normal, smooth / ruffled coat, hunched posture, reduced grooming, weight loss), signs of neurologic disease (weak grip, lethargy, ataxia, paralysis), and behavior (normal, subdued when stimulated, unresponsive). The animals on this protocol were euthanized by CO_2_ inhalation followed by cervical dislocation when they met criteria for euthanasia or at the study endpoint (24 dpi). Necropsies of mock-infected controls and DTV-infected mice were performed under ABSL-3 conditions. Harvested tissues were fixed at room temperature in 10% neutral-buffered formalin for 72 hours (with one formalin exchange after 24 hours) prior to removal from the ABSL-3 facility.

### RNA extractions and q-RT-PCR

When mice reached the moribund stage they were euthanized as described above, and the following organs were harvested and stored in TRIzol Reagent (Invitrogen, Life Technologies) according to the manufacturer’s recommendations: spleen, kidney, popliteal lymph node, testis, brain, and sciatic nerve. Blood was collected via retro-orbital bleed or terminal cardiac puncture and stored in TRIzol LS Reagent (Invitrogen, Life Technologies). Harvested tissues were homogenized using sterile stainless-steel beads in a TissueLyser II (Qiagen) for 5 minutes at a frequency of 30 Hz. Tissue and blood RNA extractions were performed using a combination of TRIzol/TRIzol LS reagent and Qiagen RNeasy mini protocols, as we have previously demonstrated that these combined protocols inactivate virus and yield high-quality RNA [22]. Briefly, chloroform was added to the tissue homogenate at a volume recommended by the TRIzol/TRIzol LS reagent protocol. The samples were mixed vigorously for 15 seconds, incubated at room temperature for 3 minutes, and centrifuged at 12,000 x g for 15 minutes at 4°C. The upper aqueous phase was collected, and one volume of 70% ethanol was added to each sample and mixed by pulse-vortexing. Each sample was then applied to a RNeasy Plus Mini column (Qiagen), and the kit’s spin protocol was followed. Total RNA was eluted from each spin column by adding 30 μL of nuclease-free water. RNA quantity and quality was detected with a DS-11+ spectrophotometer (Denovix).

Absolute quantification of viral loads in mouse tissue and blood were determined by quantitative reverse transcription real-time PCR (q-RT-PCR) using forward (5’-GCATGGTCGGATGAACAGAA-3’) and reverse (5’-CATTGGCCTTTCAGGTGTCT-3’) primers specific for the DTV NS5 gene. RNA was extracted from DTV stock of known titer that was used to infect mice in this study. Serial dilutions were made from the resulting RNA, and a linear equation was generated by plotting the cycle threshold (Ct) values of the standard curve against the corresponding log titer. Viral load Ct values from the organ samples were determined by q-RT-PCR and converted to Log_10_ FFU equivalents using the linear equation determined for the standard curve, as previously described [16]. Briefly, a standard amount of RNA was added to each well of a BioRad 96-well plate. 10 μM of q-RT-PCR forward and reverse primers specific for the DTV NS5 gene were added to each well and mixed with reagents from the iTaq Universal SYBR Green One-Step kit in a 20 μL total reaction volume (BioRad). Plates were sealed and run on a CFX96 real-time PCR platform (BioRad) with the following cycling protocol: 10 minutes at 50°C; 1 minute at 95°C; 10 seconds at 95°C followed by 30 seconds at 60°C for 45 cycles; and an 81-cycle (+0.5°C/cycle) 55–95°C melt curve.

### Histology and RNA in situ hybridization

Brain and sciatic nerve were harvested at necropsy under ABSL-3 conditions, fixed in 10% neutral buffered formalin for 72 hours, dehydrated with a standard ethanol series, and embedded in paraffin. The formalin-fixed paraffin-embedded (FFPE) brain and sciatic nerve tissues were sectioned (5 μm thickness) and dried at 34°C for 48 hours. Immediately prior to staining, each slide was baked at 60°C for 1 hour to melt the paraffin. Hematoxylin and eosin (H&E) staining of brain sections was performed following routine procedures by commercial services provided through HistoWiz (HistoWiz Inc.).

RNA *in situ* hybridization (RNA ISH) was performed with a manual RNAscope 2.5 chromogenic assay for FFPE tissues (Advanced Cell Diagnostics) according to the manufacturer’s recommendations. In this system, RNA-specific probes are hybridized to an RNA target. The target probe is then bound to a cascade of signal amplification molecules, followed by the binding of a horseradish peroxidase (HRP)-conjugated label probe or an alkaline phosphatase (AP)-conjugated label probe, resulting in signal detection upon the addition of the chromogenic substrate. Briefly, the tissue sections were deparaffinized and endogenous peroxidases were quenched with hydrogen peroxide for 10 minutes. The slides were boiled for 15 minutes in RNAscope Target Retrieval Solution and then incubated at 40°C for 30 minutes with RNAscope Protease Plus reagent. Brain and sciatic nerve sections then underwent probe hybridization with a POWV positive-sense RNA probe (catalog number 415641) followed by signal amplification and signal detection steps performed in accordance with the manufacturer’s protocol for FFPE tissue. To detect replicating viral RNA in brain tissue, a probe targeting the negative-sense complementary strand of POWV (catalog number 451341) was used. Both POWV RNAscope probes (catalog numbers 415641 and 451341) are cross-reactive with DTV RNA, and thus, do not distinguish between POWV Lineage I versus DTV Lineage II. The *Mus musculus* Duplex Ppib and Polr2a duplex probe (catalog number 321651) was included as a positive control. Slides were counterstained with a 50% solution of Gill’s hematoxylin I.

H&E and RNAscope-stained brain sections were digitally scanned with a light microscope, and the digital slide scans were examined by a board-certified veterinary pathologist contracted through HistoWiz. The pathologist performed a blinded neuro-pathological evaluation. Sagittal brain sections were examined in the rostro-caudal direction, including the olfactory bulb, cerebral cortex, hippocampus, thalamus, hypothalamus, midbrain, pons, medulla oblongata, and cerebellum, including Purkinje cell, granular cell and molecular cell layers. RNAscope staining score was graded as–(absence of staining); + (very few to low); ++ (moderate); +++ (numerous). The presence of meningitis, encephalitis or meningoencephalitis was coded as M, E or ME, respectively. Microscopic lesions (microgliosis, perivascular cuffing and/or necrosis) were graded as–(absence of lesions); + (minimal); ++ (mild); +++ (marked).

### Statistical analysis

Statistical analyses were performed using GraphPad Prism software Version 8.1.0. For survival analysis, Kaplan-Meier curves were plotted and analyzed by the log-rank test. At each time point through 8 dpi (when sample sizes are still large enough for statistical comparisons), one-way ANOVA followed by Tukey’s multiple comparisons test was used to compare percentage weight change or clinical scores between the infection doses. Dunnett’s multiple comparison test was used to compare percentage weight change or clinical scores between the controls versus infection doses. For organ viral load analyses, one-way ANOVA was used to compare viral loads between infection doses, and Dunnett’s multiple comparison test was used to compare viral loads between the controls versus infection doses. An unpaired two-tailed t-test was used to compare organ viral loads between male versus female mice within a specific infection dose.

## Results

### Peripheral inoculation of immunocompetent mice with DTV-Spooner results in dose-independent mortality

**Fig 1:**
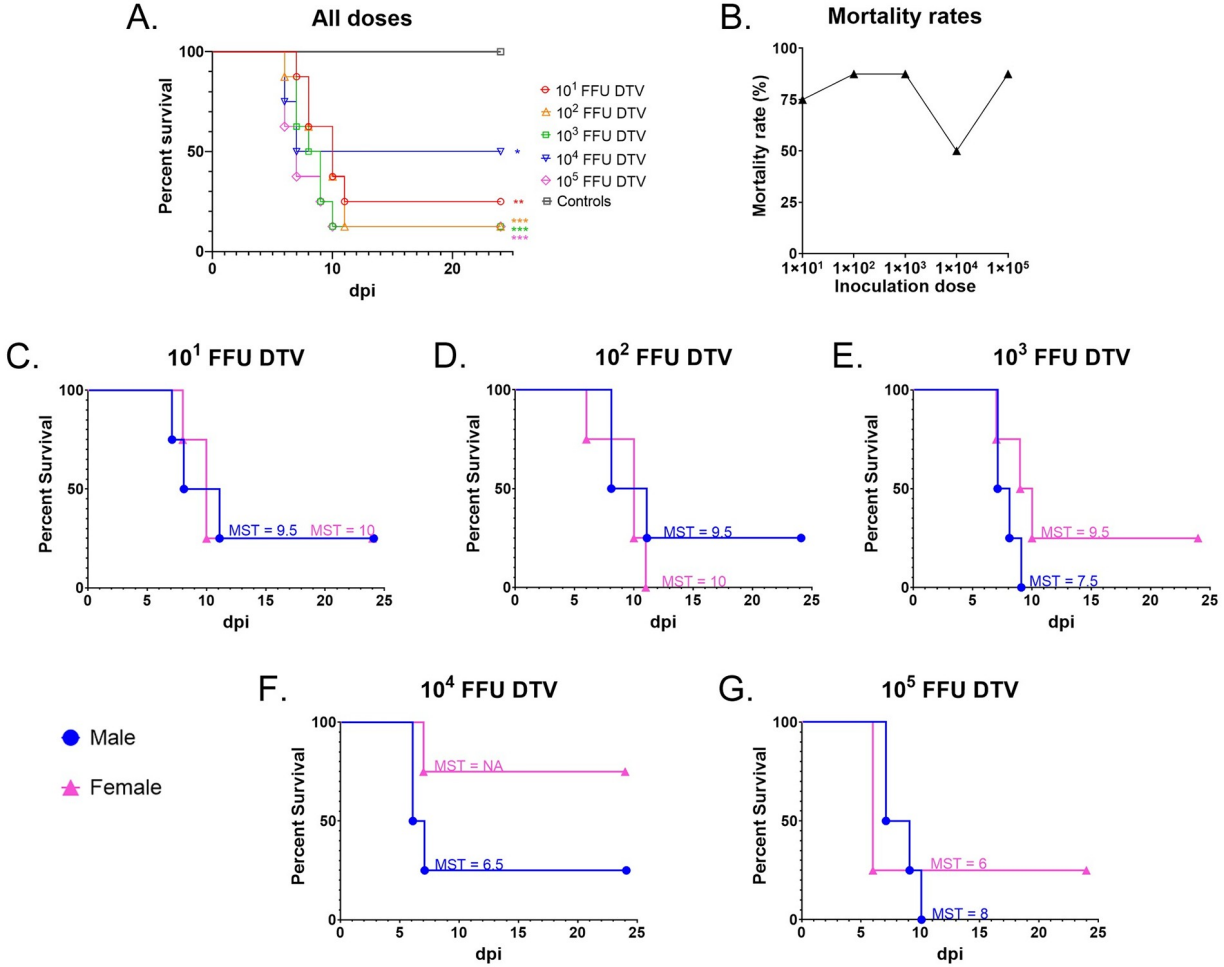
Mortality for DTV-infected mice. **A**) Kaplan-Meier survival curves for each dose cohort (n = 8 mice per dose), including mock-infected controls (n = 8). The log-rank (Mantel-Cox) test showed statistical significance for all infection doses compared to the control group. (* p < 0.05; ** p < 0.01; *** p < 0.001). The log-rank test indicated no statistically significant differences in survival between the infection doses. **B**) Mortality rates were recorded for each dose cohort (n = 8). **C–G**) Kaplan-Meier survival curves for male (n = 4) versus female (n = 4) mice inoculated with **C**) 10^1^ FFU DTV, **D**) 10^2^ FFU DTV, **E**) 10^3^ FFU DTV, **F**) 10^4^ FFU DTV, or **G**) 10^5^ FFU DTV. The median survival time (MST) in days is shown for each cohort of mice. “NA” indicates that a median survival time is undefined because greater than 50% of subjects were alive at the end of the study. All mock-infected control mice survived until the end of the study and are not graphed.

We investigated whether mortality rates directly correlate with the dose of DTV delivered to six-week-old BALB/c mice. Footpad inoculation of cohorts with sequentially increasing doses of DTV (10^1^ FFU, 10^2^ FFU, 10^3^ FFU, 10^4^ FFU, and 10^5^ FFU) did not elicit normal dose-dependent survival curves or mortality rates but instead resulted in dose-independent mortality (Fig 1A & 1B). The log-rank (Mantel-Cox) test indicated no statistically significant differences in survival between the infection doses; however, there were statistically significant differences in survival when each infection dose was compared to the control group (p = 0.0047) (Fig 1A).

We also examined the differences in survival between sexes (Fig 1C–1G). There were no statistically significant differences in mortality between infected males and infected females at any given dose, as determined by the log-rank (Mantel-Cox) test. When mice were infected with 10^1^ FFU of DTV, male and female mice both had a survival rate of 25%, with a 9.5 day median survival time (MST) for males and a 10 day MST for females. For the 10^2^ FFU infection dose, 25% of males survived (MST = 9.5 days), and no females survived (MST = 10 days). For the 10^3^ FFU dose, no males survived (MST = 7.5 days), but 25% of females survived (MST = 9.5 days). The most striking differences between sex survival rates were seen when mice were infected with 10^4^ FFU of DTV. At this dose, 25% of males survived the infection (MST = 6.5 days), while 75% of females survived. Finally, for the 10^5^ FFU dose, no males survived (MST = 8 days) and 25% of females survived (MST = 6 days).

The earliest study day that mice succumbed to disease was 6 dpi, which was documented for a 10^2^ FFU-infected female, two 10^4^ FFU-infected males, and three 10^5^ FFU-infected females. The latest study day that mice succumbed to disease was 11 dpi, which was documented for a 10^1^ FFU-infected male, a 10^2^ FFU-infected male, and a 10^2^ FFU-infected female. If an “early death” is assigned to any mice that succumb between 6–7 dpi and a “late death” is assigned to mice that succumb between 10–11 dpi, then we observed higher doses of DTV (10^3^–10^5^ FFU) causing more “early death” and lower doses of DTV (10^1^–10^2^ FFU) causing more “late deaths.”

### Morbidity following peripheral inoculation of DTV-Spooner

For the first 4 to 5 days, all DTV-infected mice remained asymptomatic and did not display outward clinical signs of disease. Weight loss was the earliest clinical sign of disease documented in this study, followed by general signs of febrile illness, including ruffled fur, reduced grooming, hunched posture, and lethargy. Ocular discharge was often observed in DTV-infected mice with reduced grooming, and sometimes resulted in complete closure of one or both eyes. Febrile signs of disease were followed by a rapid onset of neurologic illness, which ranged from weak grip strength, tremors, seizures, and either spastic or flaccid paralysis. Clinical signs of disease were assessed daily, and each mouse was assigned a cumulative clinical score based on the scoring system shown in S1 Table.

**Fig 2:**
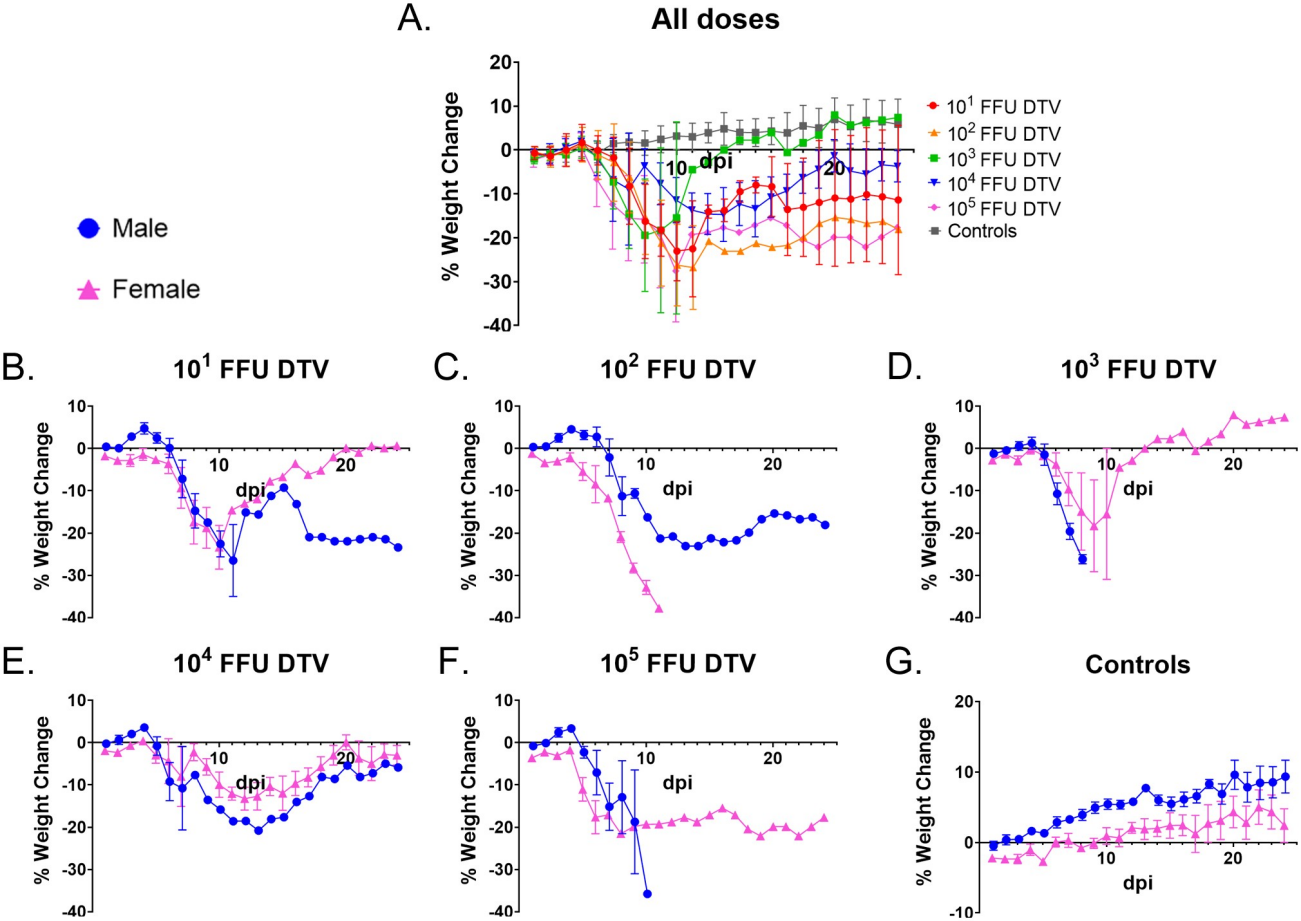
Weight loss for DTV-infected mice. Mice were weighed daily, and weights are expressed as percentage of initial body weight prior to infection (study day 0). **A**) Weight change curves for each infection dose cohort (n = 8). **B–G**) Weight was monitored daily for male (n = 4) and female (n = 4) mice inoculated with **B**) 10^1^ FFU DTV, **C**) 10^2^ FFU DTV, **D**) 10^3^ FFU DTV, **E**) 10^4^ FFU DTV, **F**) 10^5^ FFU DTV, or **G**) media. Error bars represent standard error of the mean (SEM).

**Fig 3:**
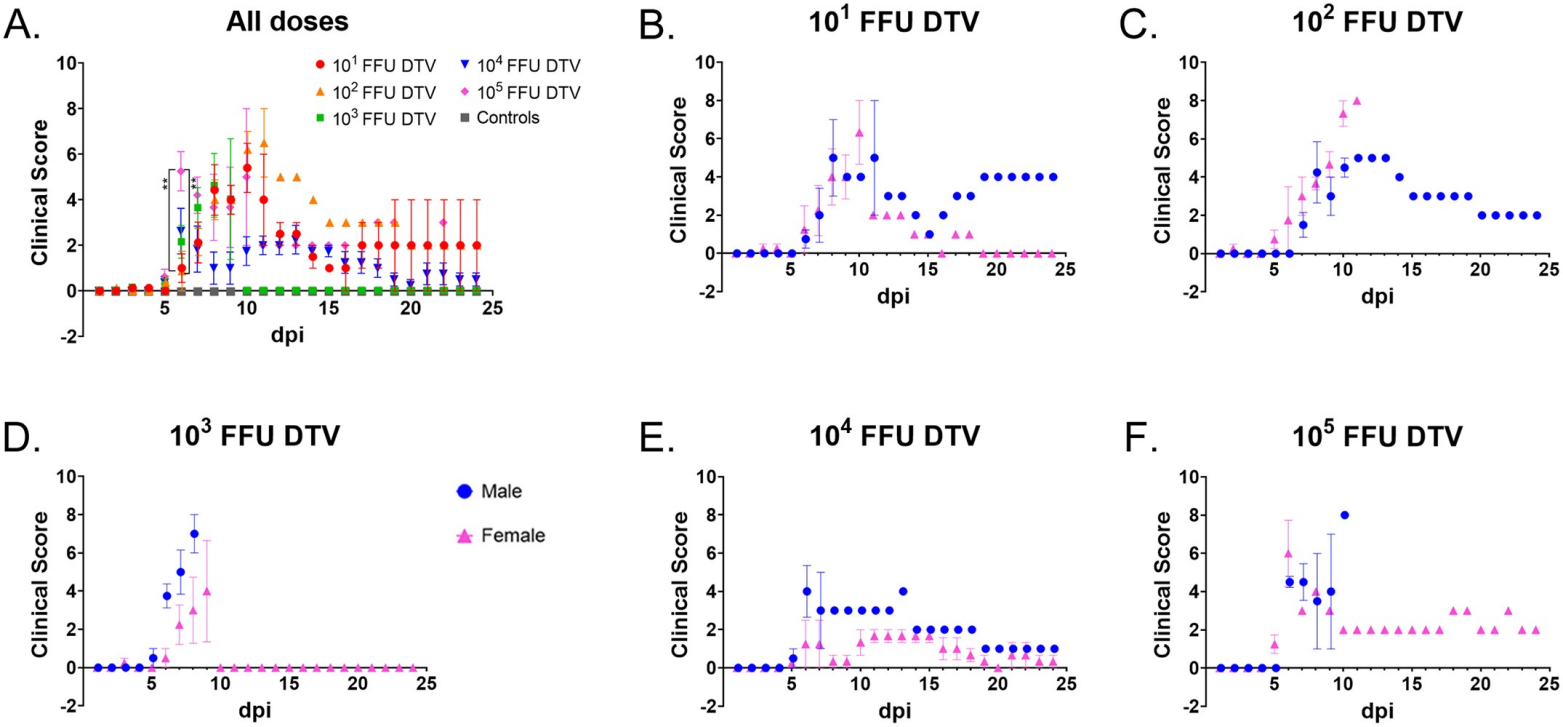
Clinical disease score for DTV-infected mice. Clinical signs of disease were assessed daily, and each mouse was assigned a cumulative clinical score based on the scoring system shown in S1 Table. **A**) Mean clinical disease scores for each infection dose cohort (n = 8) are plotted. **B–F**) Daily mean clinical disease scores are plotted for male (n = 4) and female (n = 4) mice inoculated with **B**) 10^1^ FFU DTV, **C**) 10^2^ FFU DTV, **D**) 10^3^ FFU DTV, **E**) 10^4^ FFU DTV, or **F**) 10^5^ FFU DTV. Error bars represent SEM.

At each time point through 8 dpi (when sample sizes are still large enough for statistical comparisons), one-way ANOVA followed by Tukey’s multiple comparisons test was used to compare percentage weight change or clinical scores between the infection doses. There were no statistically significant differences in percent weight change between the infection doses from 1–8 dpi, as determined by a one-way ANOVA (Fig 2A). However, there were multiple statistically significant differences in percent weight change between the control group versus specific infection doses at 5–8 dpi (S2 Table; one-way ANOVA followed by Dunnett’s multiple comparisons test). When the mean clinical disease scores were compared between infection doses at each day through 8 dpi (one-way ANOVA followed by Tukey’s multiple comparisons test), the only significant differences were at 6 dpi between the 10^1^ FFU and 10^5^ FFU infection doses (p = 0.0073) and between the 10^2^ FFU and 10^5^ FFU infection doses (p = 0.0055) (Fig 3A). Similar to the weight change data, there were multiple statistically significant differences in mean clinical score between the control group versus specific infection doses at 6–8 dpi (Fig 3A and S3 Table; one-way ANOVA followed by Dunnett’s multiple comparisons test).

At 8 dpi, the average weight loss surpassed 10% for both the male and female cohorts infected with 10^1^ FFU of DTV (Fig 2B). One female and one male survived infection with 10^1^ FFU of DTV, and the surviving female steadily gained weight from 11 dpi to the end of the study, whereas the surviving male had a varied pattern of weight change which plateaued at ~ -22% from 17 dpi to the end of the study. The mean clinical score for 10^1^ FFU-infected female mice peaked at 10 dpi (6.33 ± 1.67, n = 3), while the mean clinical score for 10^1^ FFU-infected males peaked at 8 dpi (5.0 ± 2.0, n = 5) (Fig 3B). The one surviving 10^1^ FFU-infected male maintained a clinical score of 4 from 19 to 24 dpi, which correlates with its degree of weight loss and the presence of a head tilt (a neurological sign of disease) during that timeframe (Fig 3B and S1A Fig).

For mice infected with 10^2^ FFU of DTV, the average weight loss surpassed 10% by 7 dpi for the female cohort and 8 dpi for the male cohort (Fig 2C). The mean clinical score for 10^2^ FFU-infected female mice peaked at 10 dpi (7.33 ± 0.67, n = 3), and the mean clinical score for 10^2^ FFU-infected males peaked at 10 dpi (4.5 ± 0.5, n = 2) (Fig 3C). One male displayed weak grip strength and generalized dysfunction of a front limb but survived until the end of the study with a steadily decreasing clinical score beginning at 13 dpi.

For the 10^3^ FFU-infected mice, female average weight loss surpassed 10% by 8 dpi while the male cohort only required 6 days for the average weight loss to exceed 10% (Fig 2D). Similarly, the clinical scores for the male and female 10^3^ FFU cohorts followed the same pattern as weight change, where the mean clinical score for males peaked at 8 dpi (7.0 ± 1.0, n = 2) while the female mean clinical score peaked at 9 dpi (4.0 ± 2.65, n = 3) and was lower than that of the male cohort (Fig 3D). For the one female that survived infection with 10^3^ FFU of DTV, a clinical score of 0 was maintained from 0 dpi through 24 dpi. This mouse was indeed infected with DTV as its viremia peaked at 1 dpi at 7.39 x 10^2^ FFU equivalents/μg RNA.

The overall morbidity of the 10^4^ FFU-infected mice was unique from the other infection doses. This was the only dose where the mean % weight change for survivors from both sexes steadily approached 0 by the end of the study (Fig 2E). The lowest mean clinical score peak for any male cohort was that of the 10^4^ FFU-infected males where the mean clinical score peaked at 6 dpi (4.0 ± 1.35, n = 4) (Fig 3E). Likewise, the mean clinical score for the 10^4^ FFU-infected female cohort peaked and plateaued at 1.67 ± 1.35 (n = 3) from 11 dpi through 15 dpi and was the lowest mean clinical score peak across all female cohorts (Fig 3E). For all mice that succumbed to infection with 10^4^ FFU of DTV, the moribund disease state was preceded by a generalized febrile illness or signs of neurological involvement (tremors, paralysis of a single front or hind limb, etc.) (S1D Fig). The majority (75%) of female mice survived infection with 10^4^ FFU of DTV and showed no outward signs of disease; however, the one female in the 10^4^ FFU cohort that succumbed did so abruptly, and its moribund state was only preceded by ~ 24 hours of lethargy, ruffled coat, reduced grooming and a limp in the left hind limb (S1D Fig). The one male that survived infection with 10^4^ FFU of DTV began gaining weight at 14 dpi, which also aligns with the timeline where the clinical signs of disease lessened and then disappeared for this mouse (Figs 2E and 3E, S1D Fig).

For the 10^5^ FFU-infected mice, the average weight loss surpassed 10% by 5 dpi for the female cohort and 7 dpi for the male cohort (Fig 2F). The mean clinical score for males peaked at 6 dpi (4.5 ± 0.29, n = 4) and plateaued through 7 dpi, and the female mean clinical score also peaked at 6 dpi (6.0 ± 1.73, n = 4) (Fig 3F). For the one female that survived infection with 10^5^ FFU of DTV, a clinical score ranging from 2.0 to 3.0 was maintained from 9 dpi through 24 dpi, which is supported by this subject’s percent weight change which remained < -15% from 6 dpi through the end of the study.

### Systemic detection of viral loads in DTV-infected mice

**Fig 4:**
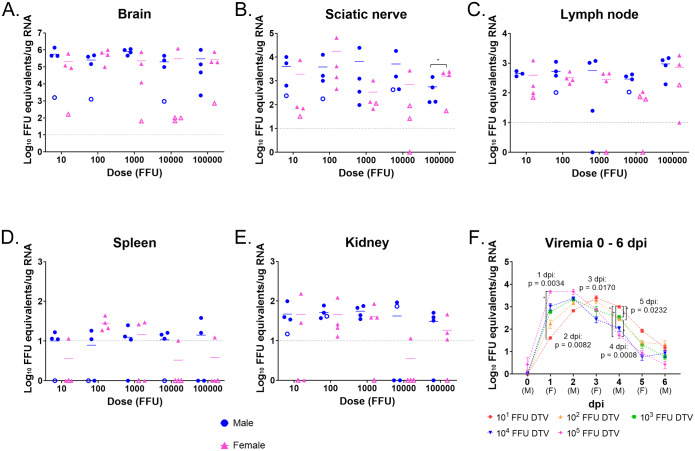
Detection of DTV in organs. **A–E**) Organs were harvested at the time of euthanasia and viral loads were analyzed via q-RT-PCR. Viral load data are expressed as FFU equivalents per microgram of RNA after normalization to a standard curve. DTV titers are plotted for each mouse tissue, and horizontal bars indicate mean values for the group. Solid symbols represent mice that succumbed to disease, while open symbols represent mice that survived until the end of the study. Statistical significance between male and female titers was determined by an unpaired two-tailed t-test. * = p < 0.05. **F**) Male and female mice were retro-orbitally bled on alternating days and blood was analyzed for viral load via q-RT-PCR. Each symbol represents the mean viremia (n = 4), and error bars are SEM. P-values were calculated by Welch’s ANOVA. Asterisk indicates the pair that shows significant difference with Dunnett’s T3 post-hoc test.

DTV infection at each inoculation dose was characterized in multiple organs of male and female mice (Fig 4). Organs were harvested when mice succumbed to disease or at the conclusion of the study (24 dpi), and q-RT-PCR was utilized to screen for the presence of DTV RNA. DTV RNA was detected in the blood, brain, sciatic nerve, lymph node, spleen, kidney, and testis in mice infected with all doses of DTV (ranging from 10^1^–10^5^ FFU). There were no statistically significant differences between infection doses for brain, sciatic nerve, lymph node, spleen, kidney, and testis viral loads as determined by one-way ANOVA. This held true when all mice were included in the analyses (n = 8 organs per infection dose) and when organs from surviving mice were removed from the analyses.

As POWV is known to be a neurotropic flavivirus in inbred laboratory mouse strains, such as BALB/c and C57BL/6, we sought to examine DTV viral loads and pathology in the nervous system of peripherally inoculated mice. There were no statistically significant differences in brain viral loads between the infection doses (one-way ANOVA); however, there was a statistically significant difference between brain viral loads for 10^3^ FFU-infected mice versus control mice (p = 0.0307, Dunnett’s multiple comparisons). Furthermore, there were no statistically significant differences (unpaired two-tailed t-test) when brain viral loads for a specific infection dose were compared between male versus female mice that succumbed. For mice that succumbed to disease, brain viral loads ranged from 10^3.32^ to 10^6.13^ FFU equivalents/μg RNA, with the mean titer equal to 10^5.68^ FFU equivalents/μg RNA. For surviving mice, the range in brain viral loads was 10^1.81^ to 10^3.20^ FFU equivalents/μg RNA (mean titer = 10^2.74^ FFU equivalents/μg RNA (Fig 4A). To determine if the viral RNA detected in the brains of surviving mice was infectious replicating DTV RNA, RNA *in situ* hybridization (ISH) of the negative-sense complementary strands of DTV was performed on brain sections from all surviving mice. Positive foci of negative-sense DTV RNA were found in multiple brain regions in every survivor’s brain, indicating permissive viral replication.

Sciatic nerve was the second nervous system tissue examined for viral load in this study. At the time of euthanasia, sciatic nerve was harvested ipsilateral to the site of DTV inoculation. For mice that succumbed to disease, sciatic nerve viral loads ranged from 10^1.82^ to 10^4.81^ FFU equivalents/μg RNA (mean titer = 10^3.73^ FFU equivalents/μg RNA) (Fig 4B). While for surviving mice, the range in sciatic nerve viral loads (above the limit of detection) was from 10^1.42^ to 10^2.63^ FFU equivalents/μg RNA. There were no statistically significant differences in sciatic nerve viral loads between the infection doses (one-way ANOVA) or between the controls versus infection doses (Dunnett’s multiple comparisons); however, when sciatic nerve viral loads were compared between male versus female mice that succumbed, the female mice infected with 10^5^ FFU of DTV had significantly higher sciatic nerve titers (mean = 10^3.32^ FFU equivalents/μg RNA) than the male mice infected with 10^5^ FFU of DTV (mean = 10^2.75^ FFU equivalents/μg RNA) (p = 0.0130, unpaired two-tailed t-test). RNA ISH for DTV viral RNA was performed on sciatic nerves from two males and two females infected with 10^4^ FFU of DTV (S2 Fig). DTV viral RNA was visualized in the sciatic nerves of both male mice, one of which succumbed at 6 dpi and the other survived until the study’s completion at 24 dpi (S2A & S2B Fig). Viral RNA was visualized in the sciatic nerve section of the female that succumbed at 7 dpi, but not in the female that survived to 24 dpi (S2C & S2D Fig). This RNA ISH data supports the sciatic nerve q-RT-PCR viral load analysis of the sciatic nerve.

DTV RNA was detected in lymphoid tissue, including the popliteal lymph node (ipsilateral to the site of DTV inoculation) and the spleen. Lymph node viral loads (above the limit of detection) ranged from a minimum of 10^1.0^ FFU equivalents/μg RNA to a maximum of 10^3.26^ FFU equivalents/μg RNA for mice that succumbed, and from 10^1.79^ to 10^2.26^ FFU equivalents/μg RNA for surviving mice (Fig 4C). There were no statistically significant differences in lymph node viral loads between the infection doses (one-way ANOVA); however, there was a statistically significant difference between lymph node viral loads for 10^5^ FFU-infected mice versus control mice (p = 0.0006, Dunnett’s multiple comparisons). There were no statistically significant differences in lymph node viral loads between male and female mice that succumbed for any infection dose (unpaired two-tailed t-test). Spleen titers were lower than lymph node titers, with nearly half of the spleen samples yielding titers below the limit of detection for DTV RNA (Fig 4D). For mice that succumbed to infection, spleen titers above the limit of detection ranged from 10^1.01^ to 10^1.64^ FFU equivalents/μg RNA. No spleen titers were above the limit of detection for mice that survived. There were no statistically significant differences in spleen viral loads between the infection doses (one-way ANOVA); however, there was a statistically significant difference between spleen viral loads for 10^2^ FFU-infected mice versus control mice (p = 0.0069, Dunnett’s multiple comparisons). There were no statistically significant differences (unpaired two-tailed t-test) when spleen viral loads within a given infection dose were compared between male versus female mice that succumbed.

Kidney viral loads were also examined in this study, and kidney viral loads above the limit of detection ranged from 10^1.03^ to 10^2.18^ FFU equivalents/μg RNA for mice that succumbed and from 10^1.0^ to 10^1.87^ FFU equivalents/μg RNA for mice that survived infection (Fig 4E). There were no statistically significant differences in kidney viral loads between the infection doses (one-way ANOVA). When kidney viral loads for the infection doses were compared to the controls, there was statistical significance between: the controls versus 10^1^ FFU-infected mice (p = 0.0359, Dunnett’s); controls versus 10^2^ FFU-infected mice (p = 0.0250, Dunnett’s); controls versus 10^3^ FFU-infected mice (p = 0.0273, Dunnett’s). There were no statistically significant differences (unpaired two-tailed t-test) when kidney viral loads within a given infection dose were compared between male versus female mice that succumbed. The testis viral loads were in a similar range to the spleen and kidney viral loads. 65% of male mice infected with DTV had detectable levels of DTV RNA in the testis, ranging from 10^1.07^ to 10^1.74^ FFU equivalents/μg RNA for mice that succumbed (S3A Fig). There were no statistically significant differences in testis viral loads between infection doses or between controls versus infection doses.

From 1 dpi through 4 dpi, DTV RNA was detected in the blood of all mice inoculated with each dose of virus. Viremias for mice infected with 10^5^ FFU of DTV peaked at 1 dpi (mean titer = 10^3.68^ FFU equivalents/μg RNA) and plateaued through 2 dpi, followed by a steady decline, where at 5 dpi, the mean viremia was slightly above 10 FFU equivalents/μg RNA (Fig 4F). At 1 dpi, Dunnett’s T3 multiple comparison post-hoc test demonstrated a significant difference in mean viremias between 10^5^ FFU-infected versus 10^1^ FFU-infected mice (p = 0.0492). The viremia curves for the 10^4^ FFU and 10^3^ FFU-infected mice were similar in that they both peaked at 2 dpi, with mean titers of 10^3.38^ FFU equivalents/μg RNA and 10^3.34^ FFU equivalents/μg RNA, respectively. By 5 dpi, the viremias had been cleared for both groups, as the mean viremias were less than 10 FFU equivalents/μg RNA. The viremia curves for the 10^2^ FFU and 10^1^ FFU-infected mice both peaked at 3 dpi, with mean titers of 10^3.28^ FFU equivalents/μg RNA and 10^3.39^ FFU equivalents/μg RNA, respectively. Interestingly, 10^1^ FFU-infected mice maintained the highest viremia of all infection doses from 3–5 dpi. Dunnett’s post-hoc test demonstrated significant differences in viremia between the following comparisons at 4 dpi (Fig 4F): 10^1^ FFU versus 10^2^ FFU (p = 0.0291), 10^1^ FFU versus 10^3^ FFU (p = 0.0380), 10^1^ FFU versus 10^4^ FFU (p = 0.0289), 10^1^ FFU versus 10^5^ FFU (p = 0.0236), and 10^2^ FFU versus 10^5^ FFU (p = 0.0300). For each DTV infection dose, viremias were clearing by 5–6 dpi. After 6 dpi, the next blood sample harvested for each mouse was taken at the time of euthanasia (ranging from 6–11 dpi for all mice that succumbed to infection, or 24 dpi for survivors). Terminal blood titers (above the limit of detection) ranged from 10^1.04^ to 10^1.63^ FFU equivalents/μg RNA, with over half of the terminal blood titers equal to 0 (S3B Fig). All mice that survived infection with DTV were not viremic at 24 dpi when terminal blood samples were screened for viral RNA (S3B Fig).

### Neuropathology in DTV-infected mice

Although not statistically significant, there was a striking difference in mortality and morbidity between male and female mice infected with 10^4^ FFU of DTV; therefore, a detailed neuropathological analysis was conducted for mice representative of the 10^4^ FFU infection dose in order to determine whether sex-specific pathologic patterns exist. The 10^3^ FFU infection dose was also included for neuropathological analysis since it was the median dose administered in the present study. A section from each representative brain sample underwent H&E staining while a second section was stained for viral RNA via RNA ISH. A blinded microscopic neuropathological analysis was performed for 10 mouse brains (Table 1). The distribution and severity of histologic lesions and detection of viral RNA in different regions of the brain (olfactory bulb, cerebral cortex, hippocampus, thalamus, hypothalamus, midbrain, pons, medulla oblongata, and cerebellum, including the Purkinje cell, granular cell, and molecular cell layers) are presented according to semi-quantitative scores in Table 1.

**Table 1:** Distribution and severity of histologic lesions and detection of viral RNA in the brain of DTV-infected mice. Sagittal brain sections were examined in the rostro-caudal direction. Each mouse was represented by an H&E-stained section as well as a section stained for viral RNA (teal signal) by RNA *in situ* hybridization. Viral RNA staining score was graded as — (absence of staining), + (very few to low), ++ (moderate), +++ (numerous). Microscopic lesions, including microgliosis (MG), perivascular cuffing (PC), and/or neuronal necrosis (NN), were graded as – (absence of lesions), + (minimal), ++ (mild), +++ (marked). The presence of meningitis, encephalitis, or meningoencephalitis was coded in the last column of the table as M, E, or ME, respectively. NP = region not present on the slide. OB = olfactory bulb, CTX = cerebral cortex, HPF = hippocampal formation, TH = thalamus, HY = hypothalamus, MB = midbrain, P = pons, MY = medulla, CB = cerebellum.

										CB			
Dose	Sample ID	OB	CTX	HPF	TH	HY	MB	P	MY	Purkinje Cells	Granule Cells	Molecular Cells	Meningitis/encephalitis
**10**^**3**^ **FFU DTV**	#17 Male H&E	+ MG	++ MG, PC, NN	+ MG	++ MG, PC, NN	+ MG, PC, NN	+ MG, PC, NN	+ MG, PC, NN	++ MG, PC	-	+ NN	+ MG	ME
	#17 Male viral RNA	+	+++	+	+++	+	+	+	+	+	+	+	
	#19 Male H&E	+ MG	++ MG, PC, NN	+ MG, PC	++ MG, PC, NN	++ MG, PC, NN	++ MG, PC	++ MG, NN	++ MG, NN	-	++ NN	+ MG	ME
	#19 Male viral RNA	+	+++	+	+++	++	+	++	++	++	++	+	
	#21 Female H&E	+ MG, NN	+++ MG, PC, NN	++ MG, NN	++ MG, NN	++ MG, PC, NN	+ MG	+ MG, NN	+ MG	-	-	-	ME
	#21 Female viral RNA	+	+++	++	+++	+	+	+	+	+	+	+	
	#22 Female H&E	-	-	-	-	-	-	-	NP	-	-	-	
	#22 Female viral RNA	-	-	-	-	-	-	-	NP	-	-	-	
**10**^**4**^ **FFU DTV**	#27 Male H&E	+ MG	+ MG	+ MG	+ MG, PC, NN	+ MG	++ MG, PC	+ MG	NP	-	+ NN	+ MG	ME
	#27 Male viral RNA	+	++	+	++	+	++	++	NP	+	+	+	
	#28 Male H&E	-	-	-	-	-	-	-	NP	-	-	-	
	#28 Male viral RNA	-	-	-	-	-	-	-	NP	-	-	-	
	#30 Female H&E	+ MG, NN	++ MG, PC, NN	+ MG, PC, NN	+ MG, NN	+ MG, PC, NN	+ MG, NN	+ MG, NN	+ MG	-	-	-	ME
	#30 Female viral RNA	++	+++	++	+++	++	++	+	+	-	+	-	
	#32 Female H&E	-	-	-	-	-	-	-	-	-	-	-	
	#32 Female viral RNA	-	-	-	-	-	-	-	-	-	-	-	
**Controls**	#43 Male H&E	-	-	-	-	-	-	-	-	-	-	-	
	#43 Male viral RNA	-	-	-	-	-	-	-	-	-	-	-	
	#48 Female H&E	-	-	-	-	-	-	-	NP	-	-	-	
	#48 Female viral RNA	-	-	-	-	-	-	-	NP	-	-	-	

**Fig 5:**
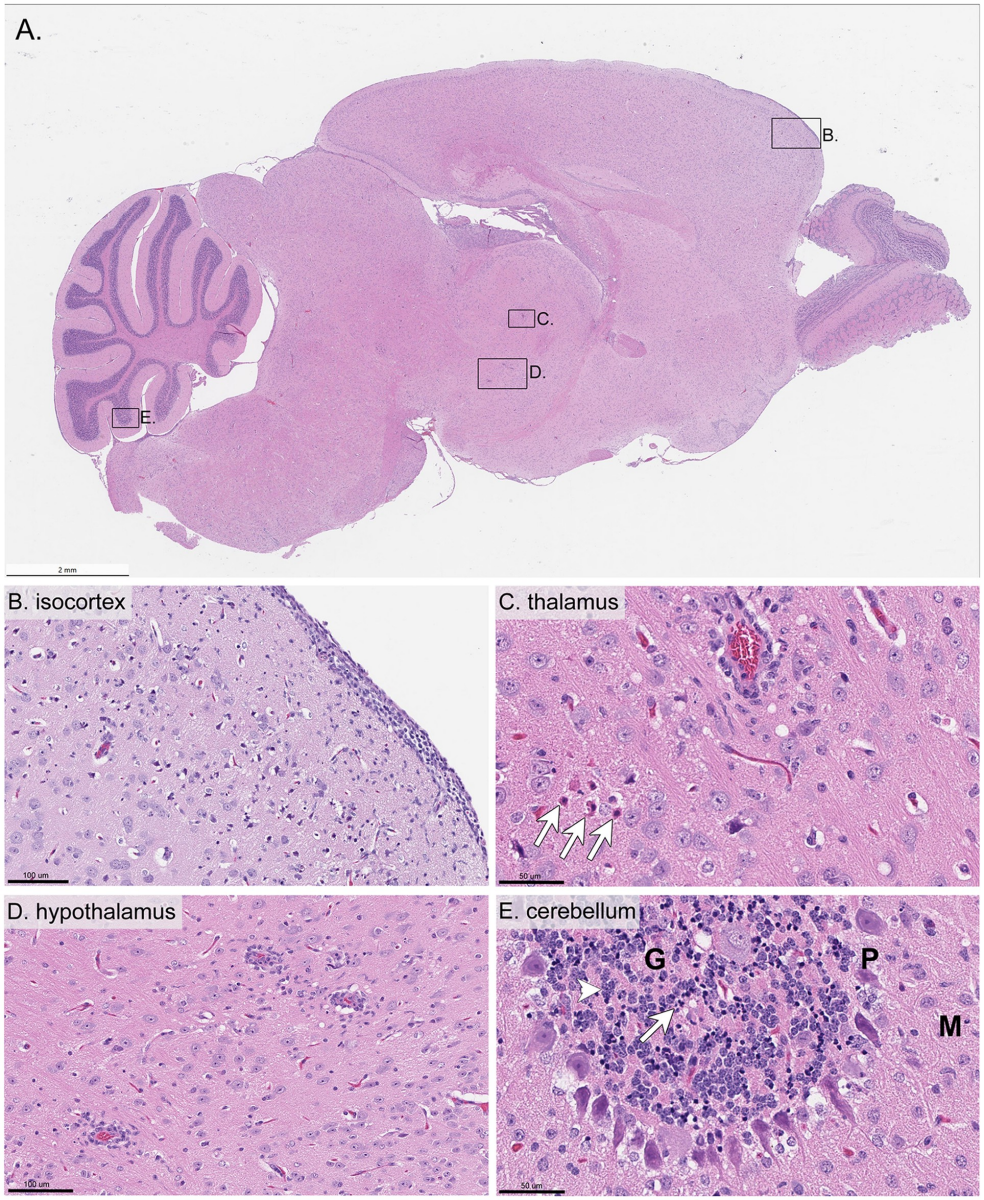
Hematoxylin and eosin-stained sections of brain from a mouse inoculated with 10^3^ FFU DTV. This tissue was harvested from a moribund mouse at 7 dpi and H&E stained. **A**) Whole brain sagittal cross-section. **B**) Isocortex **C**) Thalamus (arrows indicate eosinophilic degenerating neurons characterized by nuclear pyknosis) **D**) Hypothalamus **E**) Cerebellum (arrow points to basophilic and shrunken neurons, whereas the arrowhead points to adjacent more normal neurons). G = granule cell layer, P = Purkinje cell layer, M = molecular cell layer.

Five out of ten analyzed mice, including both 10^3^ FFU-infected males, one 10^3^ FFU-infected female, one 10^4^ FFU-infected male, and one 10^4^ FFU-infected female, exhibited clear microscopic lesions of meningoencephalitis. All mice that presented with meningoencephalitis succumbed to disease. These lesions were characterized by inflammation of the meninges (Fig 5B) and brain parenchyma, with microglial activation (Fig 5B & 5D) and perivascular cuffing (mononuclear cell infiltrates) (Fig 5C). In the most severely affected areas, neuronal necrosis, as evidenced by shrunken eosinophilic neurons with either pyknotic or karyorrhectic nuclei, was frequently associated with neurophil vacuolation (Fig 5B & 5C). Overall, microscopic lesions ranged from minimal to marked severity in affected regions and tended to be more conspicuous in the cerebral cortex and brainstem (Table 1). The only noticeable difference in neuropathology between the male and female mice that succumbed to disease was the lack of microscopic lesions in the cerebellum of the female mice (Table 1).

A few minimal focal microscopic changes with no correlation in viral RNA detection were observed in the three DTV-infected brains from surviving mice. These included small foci of perivascular infiltration of mononuclear cells in the cerebral cortex, pons, and thalamus in the surviving 10^3^ FFU-infected female, 10^4^ FFU-infected male, and 10^4^ FFU-infected female; however, these small foci did not constitute microscopic lesions and are therefore not reflected on Table 1. No microscopic lesions or small foci of perivascular infiltration were observed in the mock-infected control male and female brains.

**Fig 6:**
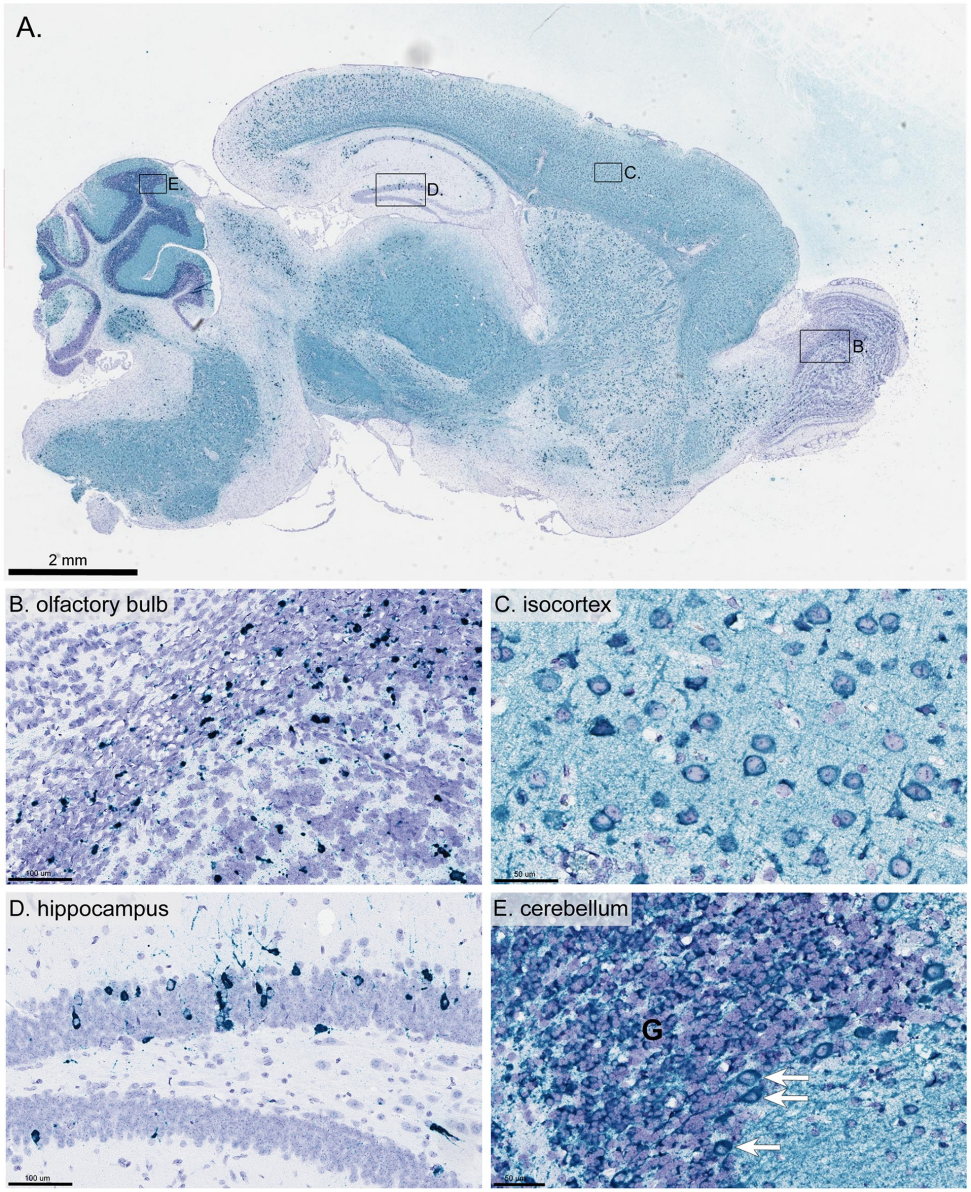
Distribution of positive-sense viral RNA in the brain of a mouse inoculated with 10^3^ FFU DTV by RNA *in situ* hybridization. This tissue was harvested from a moribund mouse at 7 dpi and stained for positive-sense POWV RNA (blue-green stain) and counter-stained with Hematoxylin (purple). **A**) Whole brain sagittal cross-section. **B**) Olfactory bulb **C**) Isocortex **D**) Hippocampus **E**) Cerebellum (arrows point to positively-stained Purkinje cells). G = granule cell layer.

In the brains of all mice that presented with meningoencephalitis, there was extensive distribution of positive-sense genomic DTV RNA (Table 1). Depending on the brain region, the positive-sense viral RNA presented with very little to low amounts of punctate dot signal to numerous dot clusters of intense signal (Fig 6 & Table 1). DTV RNA co-localized with neurons in the cerebral cortex (Fig 6C), the brainstem (including the thalamus, hypothalamus, midbrain, pons, and medulla oblongata), and the hippocampus (Fig 6D), specifically the granular cell layer of dentate gyrus and pyramidal cell layer of Ammon’s horn. Lower amounts of viral RNA were detected in the granular cell layers of the olfactory bulb (Fig 6B) and the granular cell layer, Purkinje cells, and molecular cell layer of the cerebellum (Fig 6E). Of note, most of the positively-stained neurons were histomorphologically normal, and microscopic lesions of neuronal injury were generally much less common than the positively-stained neurons. Both cerebral cortex and brainstem appear to be the most vulnerable brain regions in these DTV-infected mice.

RNA ISH detection of negative-sense complementary strand DTV RNA was performed in order to determine if DTV was replicating in the brains of surviving mice. For all surviving mice, foci of negative-sense DTV RNA were detected throughout the brain, providing clear evidence of DTV replication. S4 Fig shows representative brain regions with positive signal for the replicating complementary strand of DTV RNA in a mouse that succumbed to disease at 6 dpi (S4A Fig) and a mouse that survived until study completion at 24 dpi (S4B Fig). Foci of negative-sense DTV RNA were identified in the olfactory bulb, isocortex, hippocampus, and cerebellum (S4 Fig), indicating permissive viral replication in the brains of mice that succumbed to disease as well as those that survived the infection.

## Discussion

In North America, *I*. *scapularis* vectors several human pathogens, including *Borrelia burgdorferi*, *Borrelia mayonii*, *Anaplasma phagocytophilum*, *Babesia microti*, *Borrelia miyamotoi*, *Ehrlichia muris eauclairensis*, and POWV/DTV. Over the past several decades, there has been an accelerated rate of *I*. *scapularis*-borne pathogen discovery, coupled with an expansion in geographic range of *I*. *scapularis* in the United States [23–24]. The incidence of nationally notifiable *I*. *scapularis*-borne diseases, including Lyme disease, anaplasmosis, POWV disease, and babesiosis, has also been rising. In recent years, the incidence of POWV disease cases has increased, with a total of 188 POWV cases reported between 2004 to 2019, over half of which were reported during the period between 2016 to 2019 [7].

Few POWV disease cases are identified by lineage, and the literature is sparse when it comes to describing confirmed cases of DTV [25–30]. Because POWV and DTV are serologically indistinguishable and must be differentiated by genetic sequence analysis, an unknown portion of previously reported POWV cases may have actually been DTV cases [4, 27]. Due to the uncertainty of the etiologic agent (POWV or DTV) for the majority of previously reported human POWV disease cases, it is not known whether clinical manifestations or pathology differ between individuals infected with POWV versus DTV. Such phenomena have been well-documented for different TBEV subtypes, and it is possible that POWV and DTV may differ in their capacities to cause human disease. Comparative pathogenesis studies for POWV and DTV will help answer such questions.

Mice lend themselves well to modelling flavivirus encephalitis, largely because they can recapitulate many of the neurological disease syndromes observed in humans, and because most laboratory strains of mice are susceptible to flavivirus-induced encephalitis. Mouse models have been used to determine the tropism of tick-borne flaviviruses, to compare the pathogenicity of different virus strains, to test the efficacy of vaccines and therapeutics, and to elucidate host, viral, and experimental factors that influence disease outcome [15, 19, 31–32]. The inbred laboratory mouse strain, BALB/c, was employed for comparative pathogenesis studies of tick-borne flaviviruses, including viscerotropic and neurotropic viruses, and as a model for TBEV and POWV disease [15–16, 18, 32]. However, prior to this work, no study had comprehensively characterized the clinical disease outcome in a small animal model across a spectrum of POWV/DTV infection doses. Here, we used BALB/c mice to develop a DTV pathogenesis model that mimics the clinical manifestations of human POWV disease. We investigated the morbidity, mortality, tissue tropism, and neuropathology in six-week-old male and female mice footpad inoculated with DTV doses ranging from 10^1^ to 10^5^ FFU of DTV.

In the present study, peripheral inoculation of immunocompetent mice with DTV-Spooner resulted in dose-independent mortality, morbidity, and organ viral loads. Following footpad inoculation of BALB/c mice cohorts with sequentially increasing doses of DTV, mice did not display a typical dose-dependent mortality curve. In fact, mice infected with 10^4^ FFU of DTV, the second highest dose included in this study, displayed the lowest mortality rate (Fig 1). All mice in the study remained asymptomatic and did not display visible signs of disease for the first 4 to 5 days after DTV infection. Weight loss was the first clinical observation, and all mice that succumbed to disease lost ≥ 10% of their initial body weight. For the majority of DTV-infected mice that succumbed to disease, the moribund disease state was preceded by 1 to 2 days of a generalized febrile illness or by signs of neurological involvement, such as weak grip strength, tremors, or paralysis of a single front or hind limb (S1 Fig). The exceptions were four female mice that progressed so rapidly to the moribund disease state that no clinical signs were documented in prior days (S1 Fig). Across all infection doses of DTV, there was no pattern of right or left hemiplegia, nor was a certain dose more prone to tremors, seizures, or paralysis. Such neurological signs of disease were observed for all infection doses in this study. Overall, the clinical observations for the DTV-infected mice mimic the clinical manifestations of POWV disease in humans.

Terminal DTV titers were characterized in multiple tissues for each infection dose of mice. Viral RNA was detected in the blood, brain, sciatic nerve, lymph node, spleen, kidney, and testis in mice infected with all doses of DTV (Fig 4). The only tissue for which there was a significant difference in viral titers between infection doses was the blood. At 1 dpi, the mean viremia of 10^1^ FFU-infected mice was significantly lower than the mean viremia of 10^5^ FFU-infected mice, while at 4 dpi, the mean viremia for 10^1^ FFU-infected mice was significantly greater than the mean viremias for all other infection doses of mice (Fig 4F). These findings highlight the early peak in viremia for mice infected with the highest dose of DTV (10^5^ FFU) compared to the delayed course of viremia observed in mice infected with the lowest dose (10^1^ FFU), and a similar pattern has been previously reported for BALB/c mice infected with POWV (LB strain) [16]. The mean terminal brain titer for all mice that succumbed to disease in the present study was 10^5.68^ FFU equivalents/μg RNA, which is comparable to the terminal brain titers observed in previous studies when four or six-week old BALB/c mice were footpad or intraperitoneally inoculated with POWV (LB strain) [16, 18]. Additionally, all surviving mice in this study had detectable levels of positive-sense genomic viral RNA in the brain at 24 dpi, which has also been shown in a C57BL/6 model of POWV (LB strain) [18]. RNA ISH detection of the negative-sense complementary strand of DTV RNA, indicative of replicating virus, showed positively stained foci in the brains of all surviving mice (S4 Fig). These findings indicate persistence of infectious DTV in the brains of surviving DTV-infected mice. Similarly, persistence of infectious West Nile virus was observed in mice with subclinical infection, demonstrating that disease is not required for flaviviruses to persist in nervous system tissues [33]. To our knowledge, the present study was the first to screen peripheral nervous system tissue for the presence of tick-borne virus RNA, and we found that the mean terminal sciatic nerve titer for mice that succumbed to disease (10^3.73^ FFU equivalents/μg RNA) was second only to the mean terminal brain titer. DTV titers in lymphoid tissues and kidney for mice that succumbed to disease were lower than the brain and sciatic nerve titers.

Histological examination of DTV-infected brains found meningoencephalitis, with microscopic lesions and positively-stained neurons most conspicuous in the cerebral cortex and brainstem (Table 1). Although DTV RNA signal was widely distributed throughout the brains of mice that succumbed to disease, most positively-stained neurons were histomorphologically normal. These findings echo previously published pathology for POWV (LB strain)-infected BALB/c mice and suggest that necrosis in the brain is not directly correlated with DTV infection [15]. Together, these findings, supported by the brain and sciatic nerve viral load data, highlight the neurotropism of DTV in this mouse model.

The National Institutes of Health now expects that sex as a biological variable will be factored into research designs, analyses, and reporting in animal studies. There is a limited body of data on whether sex is a predisposing factor to flavivirus encephalitis [31]. In several literature reviews of POWV and TBEV disease, more cases were documented in males than females [3, 27, 34–38]; however, this may not reflect a pathological gender susceptibility but instead could suggest that behavioral patterns leading to tick exposure are different between genders. In the present study, there were no significant differences in mortality between the sexes at any infection dose of DTV. DTV neuropathology was also similar between the sexes, with the exception being the lack of microscopic lesions in the cerebellum of female mice (Table 1).

Since the 1940’s, it has been reported that mice peripherally infected with certain strains of encephalitic flaviviruses do not display typical linear dose response mortality curves [39]. More recently, several mosquito and tick-borne flavivirus studies demonstrated dose-independent mortality rates of approximately 30 to 60% for inbred laboratory strains of mice following peripheral infection doses ranging from 10^2^ to 10^6^ PFU [40–42]. Longer survival time for mice challenged with low to moderate doses of TBEV appears to be a key feature of dose-independent mortality [42]. Furthermore, it is thought that the type-I interferon response counteracts virus replication at increasing infection doses of Japanese encephalitis virus, resulting in the infection dose-independent mortality observed in mice [43]. Here, we demonstrated dose-independent mortality and viral loads for mice peripherally inoculated with DTV-Spooner strain. Specifically, the second-highest infection dose (10^4^ FFU DTV) resulted in the lowest mortality rate, while infection doses above and below 10^4^ FFU DTV resulted in higher mortality rates (Fig 1). Additionally, there were no significant differences between infection doses for terminal brain, sciatic nerve, lymph node, spleen, kidney, and testis viral titers, which suggests that in this mouse model, virus infection and replication in tissues is not dependent on the infection dose. Similar examples where increasing infection doses of flavivirus do not result in a linear increase in mortality rate have been occasionally recorded [40–42]; however, the mechanism(s) behind the dose-independent mortality phenomenon observed for DTV has yet to be determined.

In conclusion, we developed a small animal model for DTV pathogenesis that mimics manifestations of POWV disease in humans. Disease outcome in these mice was not affected by sex, as both male and female mice were equally susceptible to neurological disease. Results, including the rapid onset of neurological signs of disease, meningoencephalitis characterized by microscopic lesions and widespread distribution of viral RNA in the brain, as well as high brain and sciatic nerve viral titers, all highlight the neurotropism of DTV. The mouse model detailed in this study displayed dose-independent mortality, which has been occasionally reported for mosquito- and tick-borne flaviviruses. Future work is necessary to identify the mechanism(s) behind the dose-independent mortality observed in this DTV mouse model. It is currently not known whether POWV and DTV differ in their capacities to cause human disease; therefore, this mouse model can serve as a tool for future comparative pathogenesis studies.

## Supporting information

S1 Fig Clinical course of disease DTV-infected mice. Male (n = 4) and female (n = 4) mice were inoculated with **A**) 10^1^ FFU DTV, **B**) 10^2^ FFU DTV, **C**) 10^3^ FFU DTV, **D**) 10^4^ FFU DTV, or **E**) 10^5^ FFU DTV, and disease signs were assessed daily until euthanasia. The percentage of each group of mice displaying the indicated clinical signs is shown.
(JPG)

S2 Fig Detection of viral RNA in the sciatic nerve determined by RNA *in situ* hybridization. Sciatic nerves from mice inoculated with 10^4^ FFU DTV were harvested at the time of euthanasia and stained for positive-sense POWV RNA (blue-green stain) and counter-stained with Hematoxylin (purple). **A**) Male mouse euthanized at 6 dpi. **B**) Male mouse that survived until end of study. **C**) Female mouse euthanized at 7 dpi. **D**) Female mouse that survived until end of study.
(JPG)

S3 Fig Detection of DTV in testis and terminal blood. **A & B**) Tissues were harvested at the time of euthanasia and viral loads were analyzed via q-RT-PCR. Viral load data are expressed as FFU equivalents per microgram of RNA after normalization to a standard curve. DTV titers are plotted for each mouse and horizontal bars indicate mean values for the group. Solid symbols represent mice that succumbed to disease, while open symbols represent mice that survived until the end of the study.
(JPG)

S4 Fig Distribution of negative-sense complementary strand viral RNA in the brains of mice inoculated with 10^4^ FFU DTV by RNA *in situ* hybridization. Brains were harvested and stained for negative-sense POWV RNA (blue-green stain) and counter-stained with Hematoxylin (purple). **A**) Male mouse infected with 10^4^ FFU DTV that was euthanized at 6 dpi. **B**) Male mouse infected with 10^4^ FFU DTV that survived until end of study. Arrows point to positive signal for negative-sense viral RNA.
(JPG)

S1 Table Clinical scoring criteria for DTV-infected mice. Clinical signs of disease were assessed daily, and each mouse was assigned a cumulative clinical score based on appearance, neurological signs of disease, and behavior.
(XLSX)

S2 Table Statistical significance of mean weight change when the control group is compared to an infection dose. Statistically significant differences in mean percent weight change were compared by a one-way ANOVA followed by Dunnett’s multiple comparisons test to compare the infection doses to the control group.
(XLSX)

S3 Table Statistical significance of mean clinical disease score when the control group is compared to an infection dose. Statistically significant differences in mean clinical disease scores were compared by a one-way ANOVA followed by Dunnett’s multiple comparisons test to compare the infection doses to the control group.
(XLSX)
