# Supplementary figures and images for: Development of a small animal model for deer tick virus pathogenesis mimicking human clinical outcome
Source: PLoS Negl Trop Dis. 2020 Jun 15;14(6):e0008359. doi: 10.1371/journal.pntd.0008359 (PMC7316340; doi:10.1371/journal.pntd.0008359)

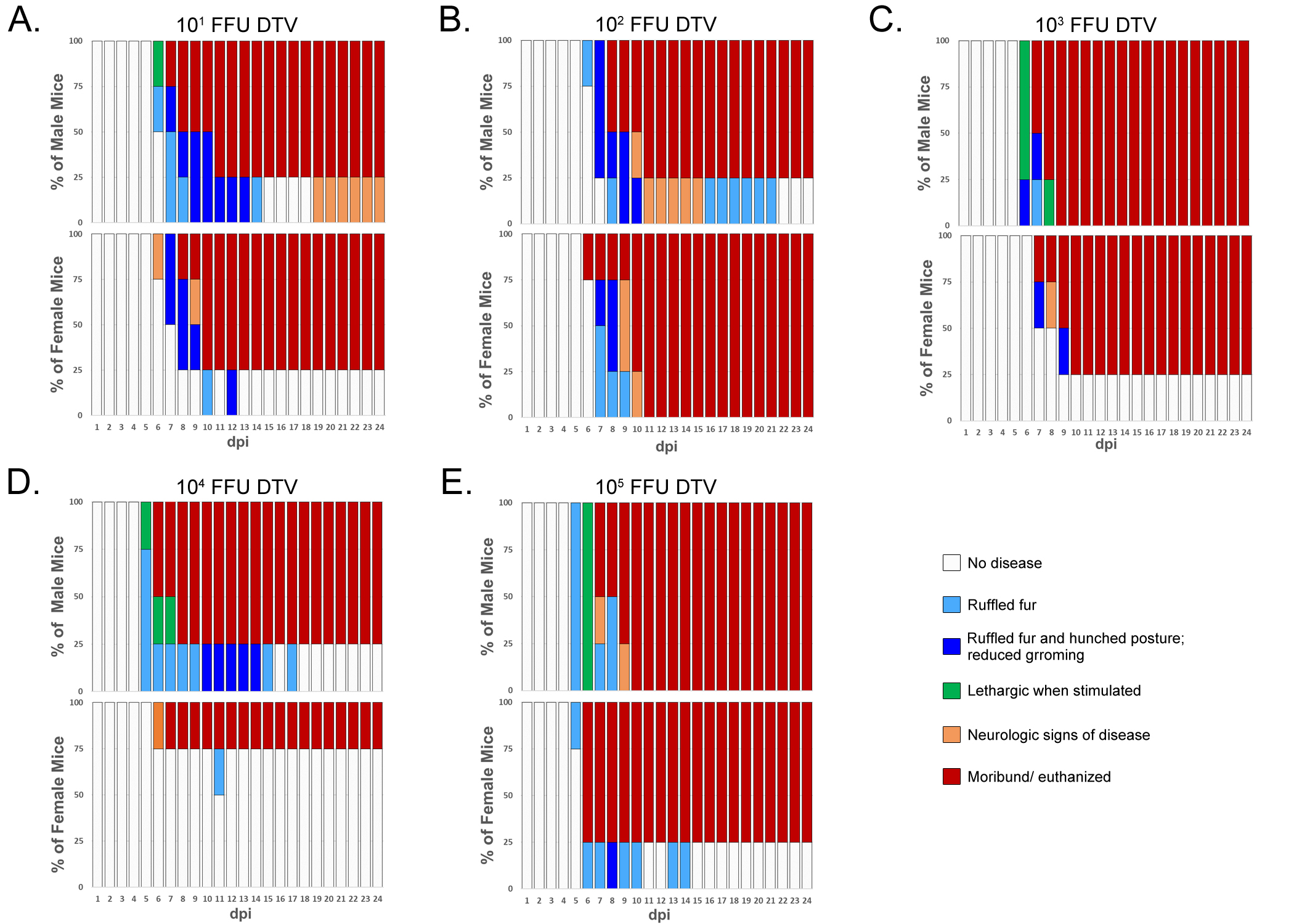

Supplement: S1 Fig — Male (n = 4) and female (n = 4) mice were inoculated with A) 101 FFU DTV, B) 102 FFU DTV, C) 103 FFU DTV, D) 104 FFU DTV, or E) 105 FFU DTV, and disease signs were assessed daily until euthanasia. The percentage of each group of mice displaying the indicated clinical signs is shown. (JPG) [file pntd.0008359.s001.jpg]

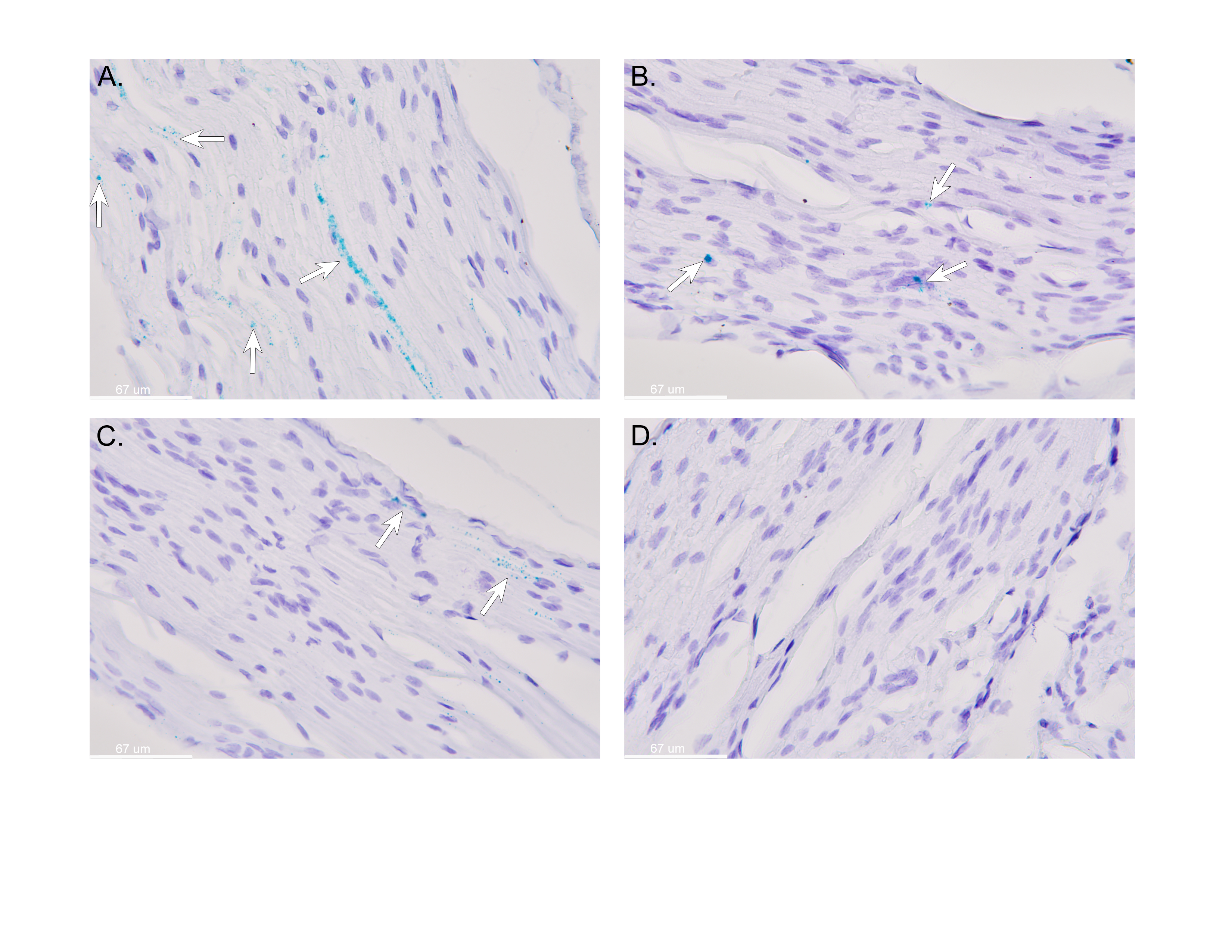

Supplement: S2 Fig — Sciatic nerves from mice inoculated with 104 FFU DTV were harvested at the time of euthanasia and stained for positive-sense POWV RNA (blue-green stain) and counter-stained with Hematoxylin (purple). A) Male mouse euthanized at 6 dpi. B) Male mouse that survived until end of study. C) Female mouse euthanized at 7 dpi. D) Female mouse that survived until end of study. (JPG) [file pntd.0008359.s002.jpg]

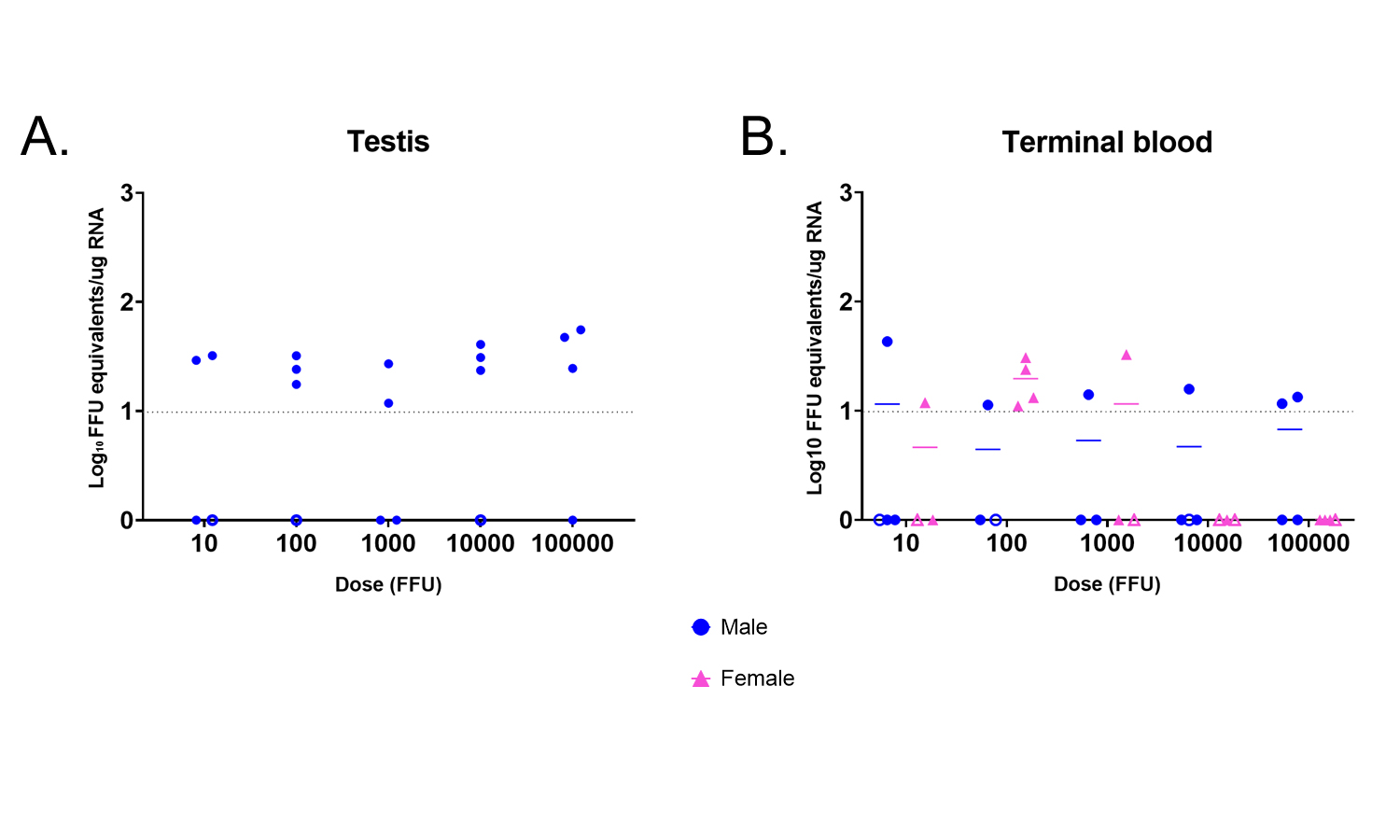

Supplement: S3 Fig — A & B) Tissues were harvested at the time of euthanasia and viral loads were analyzed via q-RT-PCR. Viral load data are expressed as FFU equivalents per microgram of RNA after normalization to a standard curve. DTV titers are plotted for each mouse and horizontal bars indicate mean values for the group. Solid symbols represent mice that succumbed to disease, while open symbols represent mice that survived until the end of the study. (JPG) [file pntd.0008359.s003.jpg]

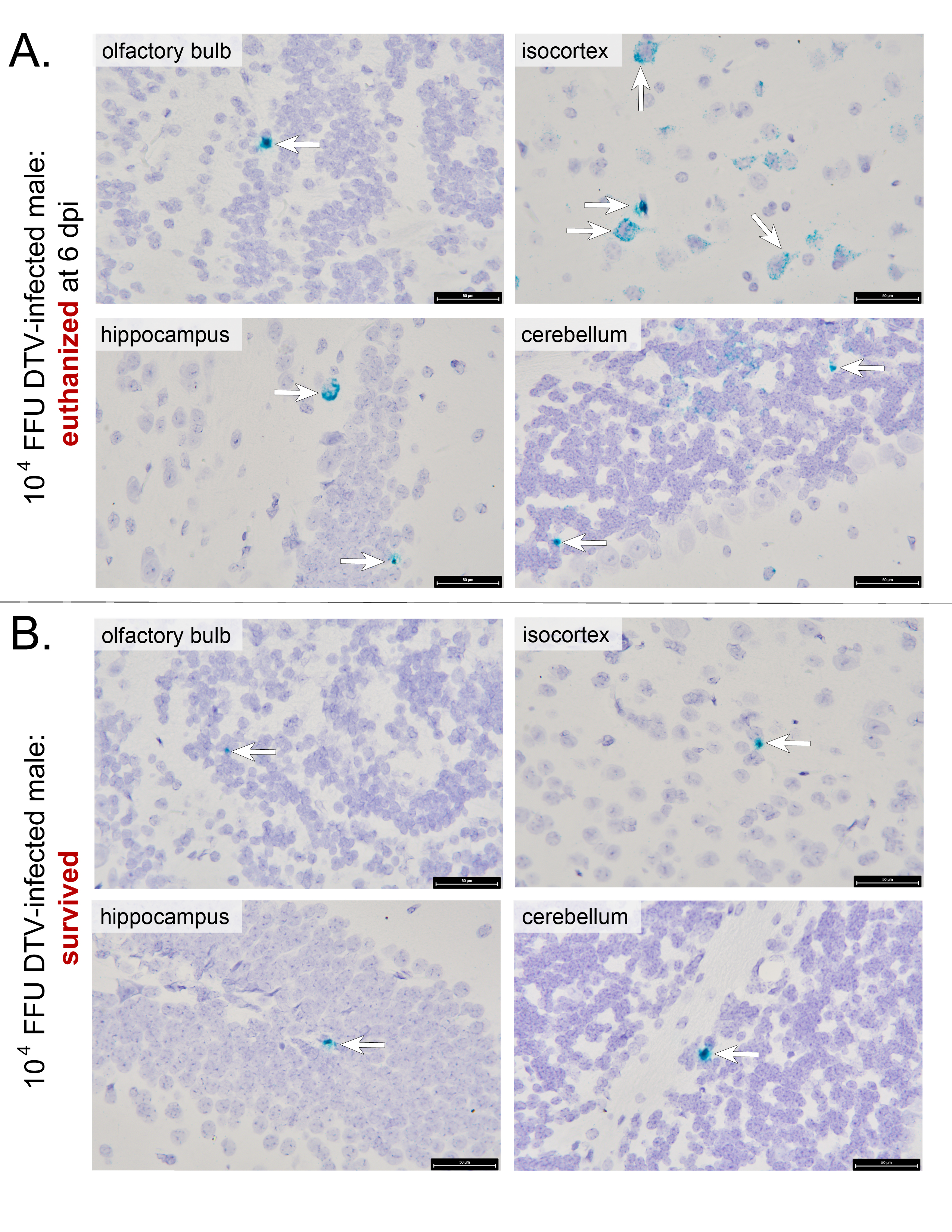

Supplement: S4 Fig — Brains were harvested and stained for negative-sense POWV RNA (blue-green stain) and counter-stained with Hematoxylin (purple). A) Male mouse infected with 104 FFU DTV that was euthanized at 6 dpi. B) Male mouse infected with 104 FFU DTV that survived until end of study. Arrows point to positive signal for negative-sense viral RNA. (JPG) [file pntd.0008359.s004.jpg]
